# Cleaning Files

**Published:** 1848-01

**Authors:** 


### CLEANING FILES.

Files used by Dentists for operating, should be of the best quality, as this item alone becomes an object of considerable expense. The teeth of the file soon become clogged or choked, by minute particles of the osseous substance, which is retained between them. They may be cleaned, from time to time, and rendered nearly as good as new, by immersing them, from twelve to twenty-four hours, in dilute muriatic acid, which should be just strong enough to act slightly upon the steel. The acid acts upon the earthy portions of the osseous substance, and separates it from the animal matter. This process being complete, the files are then taken out, washed, and boiled in water for fifteen minutes. They should then be brushed with a stiff brush until dry, when a slight coat of oil should be given them, and be placed in an oven to dry, when they are ready for use.—St. L. Ed.

